# Revealing transient events of molecular recognition via super-localization imaging of single-particle motion

**DOI:** 10.1038/s41598-019-41239-5

**Published:** 2019-03-19

**Authors:** Qing-Ying Kong, Fan Yang, Juan Song, Yi-Fan Ruan, Shan-Shan Li, Zhao-Shuai Gao, Bin Kang, Hong-Yuan Chen, Jing-Juan Xu

**Affiliations:** State Key Laboratory of Analytical Chemistry for Life Science and Collaborative Innovation Center of Chemistry for Life Sciences, School of Chemistry and Chemical Engineering, Nanjing University, 163 Xianlin Road, Nanjing, 210023 P. R. China

## Abstract

Molecular recognition plays an important role in biological systems and relates to a wide range of applications in disease diagnostics and therapeutics. Studies based on steady state or ensemble analysis may mask critical dynamic information of single recognition events. Here we report a study of monitoring the transient molecular recognition via single particle motion. We utilized a super-localization imaging methodology, to comprehensively evaluate the rotational Brownian motion of a single nanoparticle in spatial-temporal-frequential domain, with a spatial accuracy ~20 nm and a temporal resolution of ~10 ms. The transient moment of molecular encountering was captured and different binding modes were discriminated. We observed that the transient recognition events were not static states of on or off, but stochastically undergoes dynamical transformation between different binding modes. This study improves our understanding about the dynamic nature of molecular recognition events beyond the ensemble characterization via binding constant.

## Introduction

Molecular recognition is a type of specific molecular interaction between host and guest molecules and plays important roles in biological systems^1^. The nature of such process is vital important since it offers mechanisms to regulate molecular binding in biological systems, which related to a wide range of disease diagnostics and therapeutics^2–4^. This transient interaction between the host and guest may not be simply considered as static states of on or off, it probably undergoes a complex dynamic process. The accurate understanding of molecular recognition events needs to be beyond the static snapshots of single frame between the guest and host. Assembled methodologies like nuclear magnetic resonance (NMR)^5^ and polarized fluorescence spectroscopy^6^, etc., provided statistical results based on large number of molecules, however, it might mask important dynamic nature of single recognition events. Single molecular techniques, like single molecule fluorescence spectroscopy^7^ and single molecule force spectroscopy^8,9^, provide powerful tools for studying molecular interaction at single molecule level, with very high spatial resolution. However, the photo bleaching, quenching and blinking nature of fluorescence dyes make it hard to monitor the full process of interaction in long term. Single molecular force spectroscopy typically requires to connect host or guest to the tip and keep another one on the substrate, under such condition, the whole encountering process is fully controlled by the mechanical motion of the tip, thus it might miss some information when the process occurred naturally^8^. The transient events of molecular recognition required a methodology to seek molecular encountering on long time scale, and also have high spatial and temporal resolution to capture the transient moment of single encountering events.

Owing to the unique plasmon resonance properties, novel metal^10,11^ (e.g. gold or silver) nanoparticles have excellent photostability for long term analysis, and could serve as a potential tool to probe molecular interactions at single particle level. Information about molecular interactions could be obtained from either spectroscopic changes of single nanoparticle^12,13^ or changes on its motion state^14,15^. Particularly, the translational and rotational motion of a single anisotropic nanoparticle (e.g. gold nanorods) in liquid, termed rotational Brownian motion (RBM)^16–19^, was very sensitive to their surrounding environment. Through analyzing the motion state, including translation and rotation, many information about the surrounding medium and interaction between molecules and nanoparticles could be extracted. Several optical techniques, including differential interference contrast microscopy^20^, dual channel polarization dark-field microscopy^14,21^ and photothermal orientation imaging^22^, have been established to determine the orientational information of single gold nanorods in three-dimension.

Herein, we utilized a super-localization imaging methodology to comprehensively evaluate the rotational Brownian motion of single nanoparticles simultaneously in spatial, temporal and frequential domain. This method enable us to track transient molecular recognition on a single nanorod, with a spatial accuracy ~20 nm and a temporal resolution of ~10 ms. Through analyzing motion state of a single nanorod in spatial-temporal-frequential domain, the transient moment of molecular encountering was captured and different binding modes were discriminated.

## Results and Discussion

**Figure 1:**
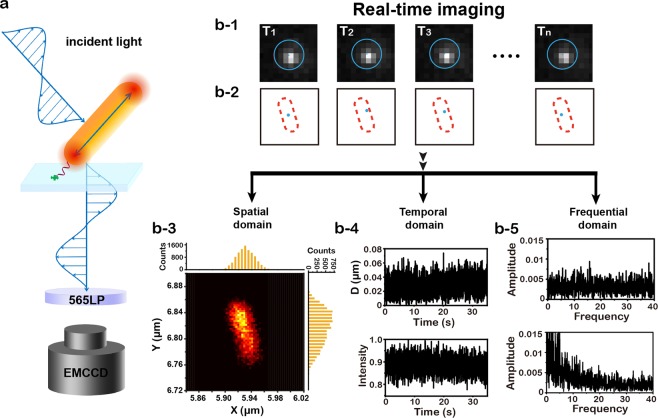
Schematic illustration of motion state tracking of a single gold nanorod (GNR) on spatial-temporal-frequential domain. (**a**) A schematic of the polarized stochastic-scattering reconstruction microscopy (PSRM). (**b-1**) Real time image sequences of a GNR. (**b-2**) spatial fluctuation of localization events at different image sequences.(**b-3**) Spatial domain: the map of localization distribution probability (LDP) of a GNR and corresponding distribution histograms at X and Y directions. (**b-4**) Temporal domain: time traces of localization dispersion D(t) and optical intensity I(t) extracted from the real tine images. (**b-5**) Frequential domain: fast Fourier transform (FFT) of optical intensity I(t) showed different rotation status.

Figure 1 showed the schematic illustration of our super localization imaging configuration. Gold nanorods (GNRs) with an average size of 40 ± 4 nm × 12 ± 2 nm (Fig. S1) were used as motion sensors here. These GNRs have a long-axis plasmon scattering band around 724 nm and a short-axis plasmon scattering around 514 nm (Fig. S2).We utilized a linear polarized grazing incidence illumination and collected the plasmon resonance scattering signal along with time. The principle of super localization imaging is based on stochastic collision of incident photons with single GNRs under low flux of incident photons (Fig. 1, Scheme S1). Briefly, the scattering signals originate from elastic collision of incident photons with GNRs. Typically, photons scattered from different positions of GNRs were collected by a camera to form an averaged photon intensity map, i.e. image. In such case, information about collision positions that hided in scattering photons was masked since the diffractive nature of photons, as a result, only a diffraction-limited image was finally observed. However, once the flux of incident photons were very low, so that only a few scatted photons were collected at one time, the collision position could be extracted from scattering image through super localization methods with spatial accuracy of 20–30 nm or even less^23^. After many frames of localization, the morphology of the GNRs could be finally remapped as a super localization image.

In our current work, high speed images were recorded by an electron-multiplying charge-coupled device (EM-CCD) at 100 frames/s, with a temporal resolution of 10 ms (Fig. 1). A 565 nm long pass filter was placed before the camera to block the short-axis scattering, as a result, only the long-axis scattering was detected. Notable that the long-axis scattering is polarization dependent, the scattering intensity reach maximum only when the nanorods long-axis is parallel to the electric field of incident light. The precise location of gold nanorods (x(t), y(t)) at a specific time (t) could be extracted by solving the optical centroid of the far field projection in each frame of image series by:

1 $${x}_{(t)}=\frac{{\iint }^{}x\mu (x,y)dxdy}{{\iint }^{}\mu (x,y)dxdy}$$

and

2 $${y}_{(t)}=\frac{{\iint }^{}y\mu (x,y)dxdy}{{\iint }^{}\mu (x,y)dxdy},$$

where *μ*(*x*, *y*) is the optical density function, which could be calculated from optical intensity I(x, y) by

3 $$\mu (x,y)=\frac{dI}{dxdy}.$$

As Fig. b-1 and b-2 revealed that the shape and brightness of the pattern and the optical centroid (i.e. precision location) varies from case by case, the position (x(t), y(t)) at each frame was defined as a localization event, therefore a series of localization events (x(t), y(t)) could be obtained after many frames of imaging. Then we defined a localization distribution probability

4 $$P=\frac{\partial N}{\partial A\partial t}=\frac{{\rm{\Delta }}N}{{\rm{\Delta }}A{\rm{\Delta }}t},$$

and reconstructed the map of localization distribution probability (LDP) in spatial domain with very high resolution, where N is the counts of localization events, A is the distribution area and Δt is the exposure time. Besides spatial domain, we also could extract time correlated function of scattering intensity I(t), translation velocity V(t) and dispersion of localization

5 $$D(t)=\sqrt{{({x}_{(t)}-\bar{x})}^{2}+{({y}_{(t)}-\bar{y})}^{2}},$$

where $(\bar{x},\bar{y})$ represents the averaged location among many frames of image stacks. The variation of D(t), V(t)and I(t) in temporal domain could be used to describe the translation and rotation of gold nanorods. Information about the rotation of nanorods in frequential domain could be further extracted by applying fast Fourier transform (FFT) to the polarization dependent I(t). All in all, the translational and rotational motion state of single nanorods could be comprehensively evaluated in spatial-temporal-frequential domains.

**Figure 2:**
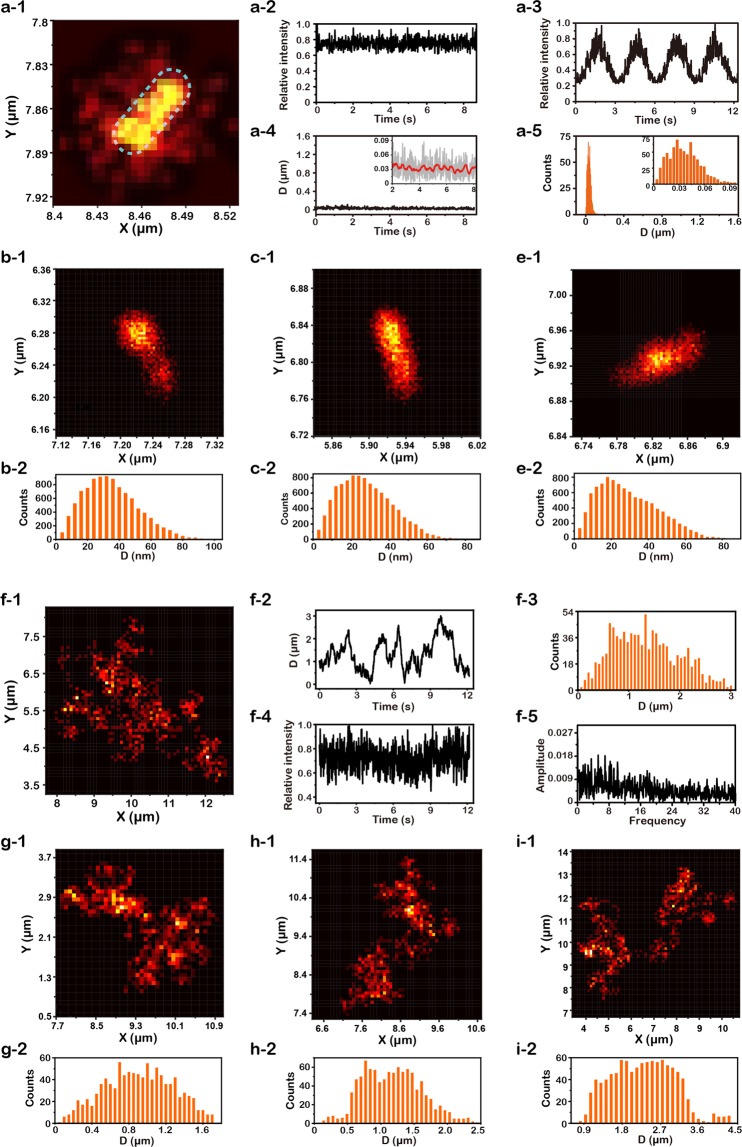
Rotational Brownian motion of a single gold nanorod (GNR) at different interface. (**a-1**~**a-5**) Super-localization imaging of an immobilized GNR at air/solid interface. (**a-1**) Localization distribution probability (LDP) map. (**a-2**) Time trace of optical intensity I(t) at fixed polarization angle. (**a-3**) Time trace of I(t) with rotational polarization angle. (**a-4**) Time traces of localization dispersion D(t). (**a-5**) Histograms of D(t).(**b-1**~**e-2**) Super-localization imaging of different immobilized GNRs at air/solid interface. (**b-1**) LDP map. (**b-2**) Histograms of D(t). (**c-1**) LDP map. (**c-2**) Histograms of D(t). (**d-1**) LDP map. (**d-2**) Histograms of D(t). (**f-1**~**f-5**) Typical rotaional brownian motion of a GNR at water/solid interface. (**f-1**) LDP map. (**f-2**) Time traces of D(t). (**f-3**) Histograms of D(t). (**f-4**) Time trace of I(t). (**f-5**) Fast Fourier transform (FFT) of I(t). (**g-1**~**i-2**) The rotaional Brownian motion of different GNRs at water/solid interface. (**g-1**) LDP map. (**g-2**) Histograms of D(t). (**h-1**) LDP map. (**h-2**) Histograms of D(t). (**i-1**) LDP map. (**i-2**) Histograms of D(t).

We firstly performed a proof of concept by monitoring a static gold nanorod deposited on the dry surface of a glass slide. In the typical LDP map as shown in Fig. 2a-1, the naonorod showed a very confined pattern about 61 nm × 26 nm, and the morphology of nanorod could be clearly distinguished. Herein, all images were taken at a fixed polarization direction parallel to nanorods long-axis. Thus we obtained a relatively homogenous map closed to the physical morphology of the GNRs. Such super localization strategy also could cooperate with a rotation polarization setup to visualize the hot spots at the tip of the GNRs, with resolution less than 20–30 nm^23^. Generally, for any optical system, the final image could be considered as the convolution result of the object (i.e. GNRs) and the point spread function. Thus, with a given size of nanorods here 40 nm × 12 nm, the spatial accuracy of our current system was calculated about 20 ± 2 nm. The scattering intensity I(t) was basically stable in long term, but with small fluctuation in short time scale (Fig. 2a-2).

**Figure 3:**
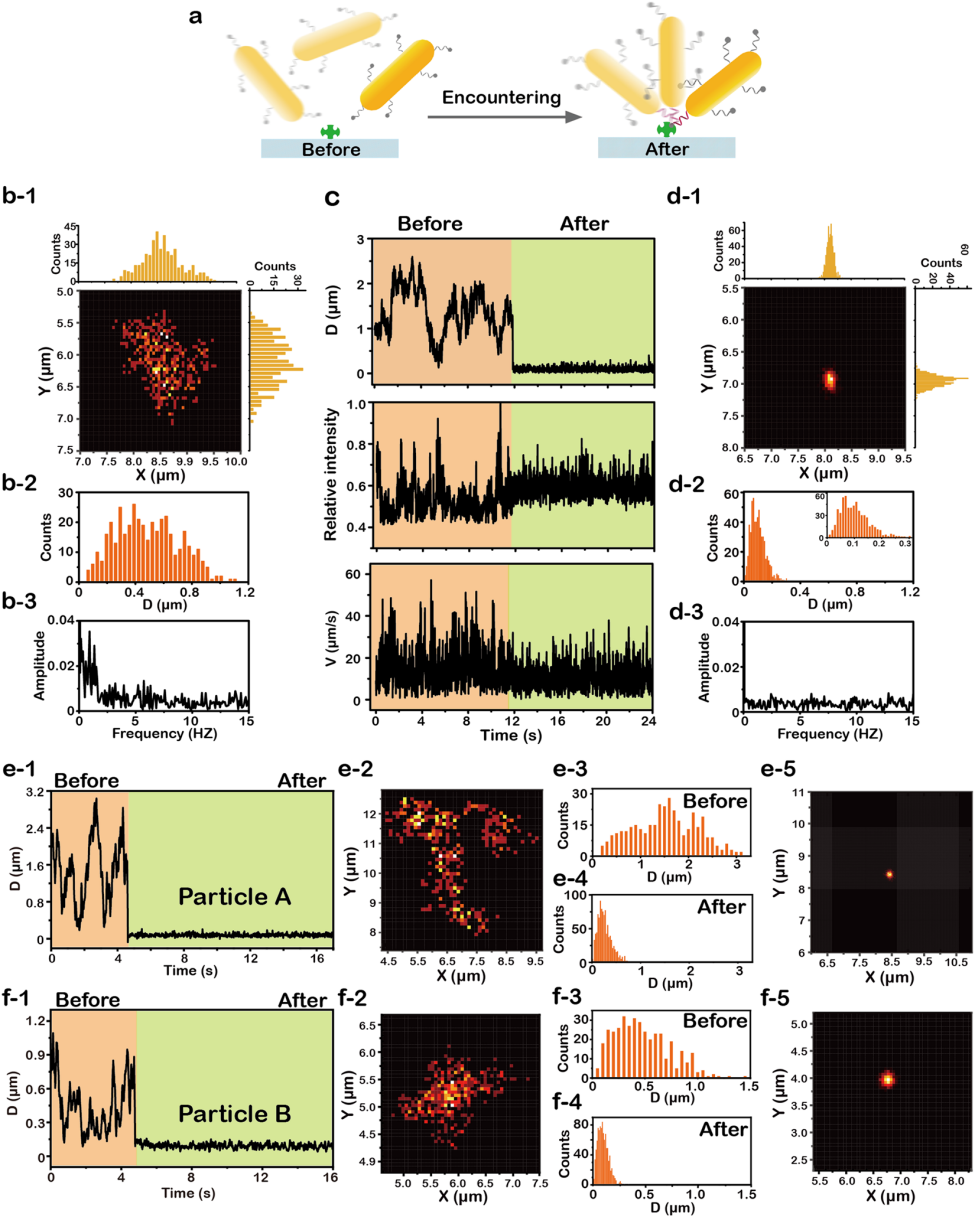
Transient encountering of molecular recognition monitored by a single gold nanorod (GNR). (**a**) Schematic illustration of biotinylated-DNA-GNR trapped by streptavidin. (**b-1**~**b-3**) Localization distribution probability (LDP) map (**b-1**), histograms of localization dispersion D(t) (**b-2**) and fast Fourier transform (FFT) of optical intensity I(t) (**b-3**) of a biotinylated-DNA-GNR before being trapped by streptavidin. (**c**) Time traces of D(t), I(t) and velocity V(t) before and after molecular encountering. (**d-1**~**d3**) LDP map (**d-1**), histograms of D(t) (**d-2**) and FFT of I(t) (**d-3**) after the biotinylated-DNA-GNR attached to the streptavidin-coated glass slide. (**e-1**) Time traces of D(t) before and after molecular encountering. (**e-2**) LDP map and (**e-3**) histograms of D(t) of Particle A(biotinlayted-DNA-GNR) before being trapped by streptavidin. (**e-5**) LDP map and (**e-4**) histograms of D(t) after the biotinylated-DNA-GNR attached to the streptavidin-coated glass slide. (**f-1**) Time traces of D(t) before and after molecular encountering. (**f-2**) LDP map and (**f-3**) histograms of D(t) of Particle B (biotinylated-DNA-GNR) before being trapped by streptavidin. (**f-5**) LDP map and (**f-4**) histograms of D(t) after the biotinylated-DNA-GNR attached to the streptavidin-coated glass slide.

**Figure 4:**
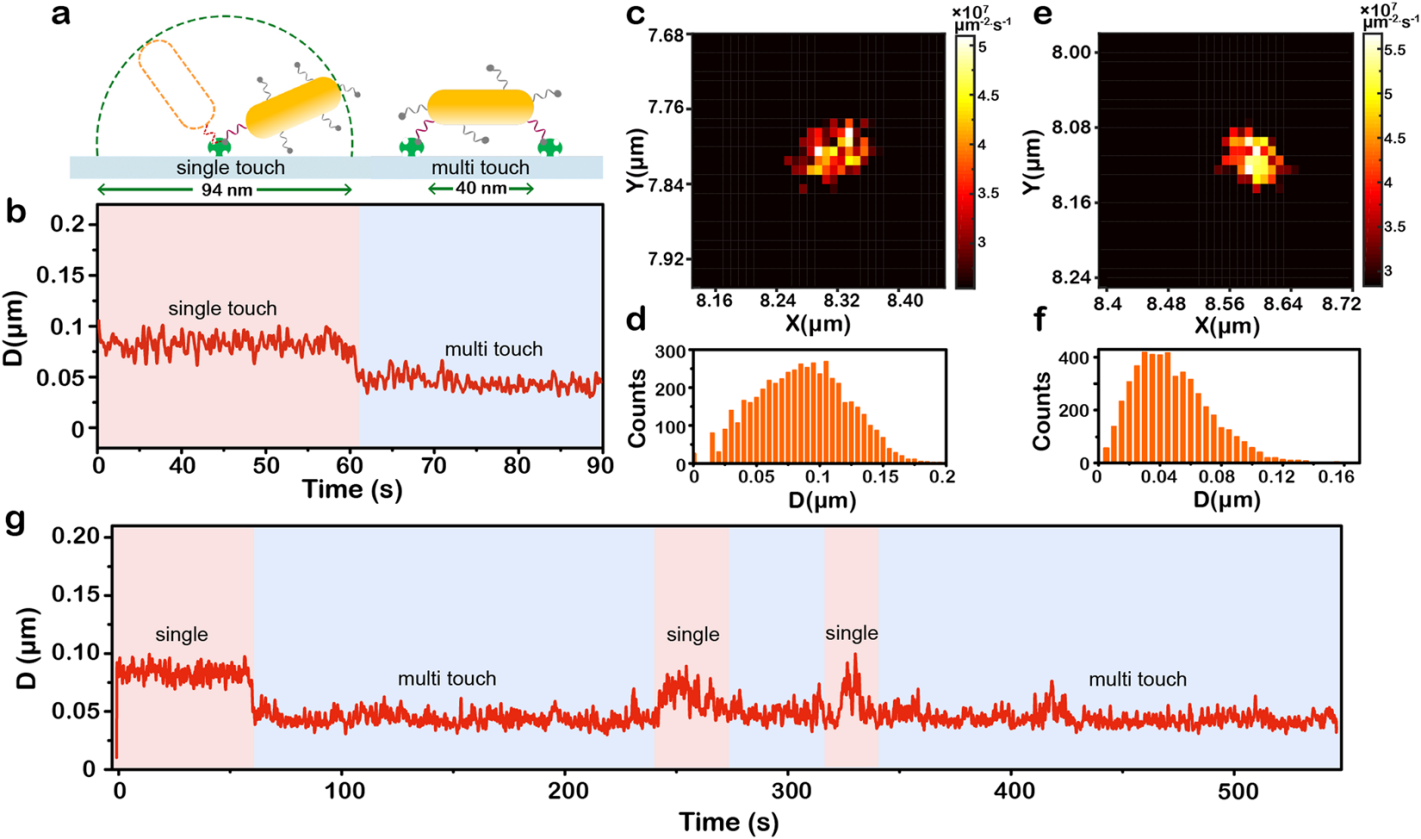
Different binding states revealed by a single gold nanorod (GNR). (**a**) Proposed model of two different binding state of biotinylated-DNA-GNR at the surface of streptavidin-coated glass slide and their motion ranges. (**b**) Time traces of localization dispersion D(t) shows the difference between the single and multi-touch. (**c**,**d**) Localization distribution probability (LDP) map (**c**) and histograms of D(t) (**d**) of single-touch state. (**e**,**f**) LDP map (**e**) and histograms of D(t) of multi-touch state. (**g**) Time traces of D(t) under a long period of observation shows conversions between single and multi-touch states.

Above results were obtained under a fixed polarization. When rotating the linear polarizer to change the angle between the nanorod long-axis and the polarized light, the scattering intensity is proportional to the square of cosine of the time (Figs 2a-3), which is consistent with previous work^24^. The dispersion of localization *D*(*t*) exhibited an average value of 35 nm with a fluctuation of ~10 nm (Fig. 2a-4,a-5). The average value of *D*(*t*) is very close to the physical size of nanorods 40 nm. It is notable that this value 35 nm is slight smaller than the physical size 40 nm, because of the anisotropic shape of the nanorods. By investigating more individual immobilized gold nanorods on the same sample, (Fig. 2b-1,c-1,e-1), and the average value of *D*(*t*) are 26 nm, 33 nm and 28 nm respectively (Fig. 2b-2,c-2,e-2). Then, we studied the typical rotational Brownian motion of a single nanorod at the water-solid interface. The nanorod translated in a region about ~3 μm (Figs 2f-1) accompanied by rotation, resulting in the large variation of *D*(*t*) and I(t) (Fig. 2f-2,f-4). The averaged *D*(*t*) of RBM in water is about 1.3 μm (Fig. 2f-3), which is much larger than the value of a static nanorod. After FFT of I(t), a wide frequency spectrum could be seen in the frequential domain, corresponding to a random rotation(Fig. 2f-5). Brownian motion of different individual nanorods at water-solid interface represent large motion region as expected. The average value of *D*(*t*) of different GNRs are 0.88 μm, 1.1 μm and 2.2 μm (Fig. 2g-2,h-2,i-2), which is 4~5 times larger than the value of immobilized nanorod. In RBM theory, the motion state of translation and rotation is a balance of thermal-driven motion and the interaction with surrounding environment^18,25^. By changing different media, the *D*(*t*) and I(t) were dramatically different (see Supporting Information for details, Fig. S3). The sensitive response of motion state to surrounding environment allows for detecting molecular interaction down to single recognition event.

With above bench mark results, we then investigated the transient encountering of molecular recognition. Biotin and streptavidin were selected as a model of molecular recognition due to their high binding constant (K = 10^13^ M^−1^). Biotin molecules were conjugated to the surface of nanorods through a 22 bases single-strand DNA (about 7 nm in length), termed biotinylated-DNA-GNRs, and the conjugation efficacy is calculated about 2 × 10^2^ per particle. The immobilization of streptavidin molecules on the glass slide are performed according to the reference^26^. Hence, we monitored the motion state of the same single gold nanorod in long term up to tens of minutes to capture the transient moment of molecular encountering. When the biotinylated-DNA-GNRs has not attached to the streptavidin-coated glass slide, their motion state is quite similar with the RBM of nanorods in water (Fig. 3b-1,b-2,b-3). Large variations of D(t), I(t) and V(t) were observed in temporal domain (Fig. 3c), corresponding to a free RBM mode. Once the biotinylated-DNA-GNRs bind to the streptavidin-coated glass slide, the motion of gold nanorods was confined to a limited region. As can be seen from the Fig. 3c, the variation in time traces of D(t), I(t) and V(t) were quite different before and after biotin-streptavidin binding, especially in the curve of D(t). Before the molecular encountering, D(t) fluctuated widely with a range up to 2.6 μm, with an averaged value about 1.4 μm. However, D(t) remains nearly constant with an average value of 95 nm after the biotinylated-DNA-GNR being trapped by streptavidin(Fig. 3d-1,d-2). The abrupt decrease on D(t) suggested a transient confinement on the translational motion of nanorods, compared with their free translation before molecular encountering (Fig. 3a). Besides the temporal domain, different motion states could also be effectively revealed by the low-frequency region of FFT spectrum (Figs 3d-3). After molecular encountering, the low-frequency region of typical RBM that corresponds to free rotation was not observed any more, which indicated the transformation from free rotation to confined rotation. Taken together, the LDP map in spatial domain, the D(t), I(t) and V(t) in temporal domain and FFT spectrum in frequential domain clearly showed the motion state change of the gold nanorod before and after molecular recognition (Fig. 3a). Same trends were observed in the time traces of D(t) of another two particles A and B (Fig. 3e-1,f-1), which also showed abrupt drop and huge difference before and after molecular recognition in the LDP map (Fig. 3e-2,e-5,f-2,f-5). These results are in good consistent with the above discussion in the example of Fig. b-1~d-3.

In order to inspect the effect of non-specific interaction, several groups of control experiments are performed. No matter the bare gold nanorods on the streptavidin-coated glass slide or the biotinylated-DNA-GNR at water/solid interface, they both move in a large region (Figs S4 and S5). Despite different nanoparticles exists variability between each other, the average D(t) are all in the micrometer scale. Consequently, the effect of non-specific interaction could be excluded and the abrupt decrease on D(t) was contributed to the encountering between the biotin and streptavidin.

On the basis of the above experiments, we found the variation of D(t) was the most remarkable parameter to distinguish different motion states. We further took deep insight into the binding modes of the biotinylated-DNA-GNRs after the molecular encountering. We propose two different binding modes of biotinylated-DNA-GNR at the surface of streptavidin-coated glass slide, named single-touch and multi-touch (Fig. 4a). These two binding modes theoretically resulted in different motion states, which could be distinguished by monitoring the variation of D(t). After molecular encountering, D(t) remained almost constant (83 ± 5 nm) under a long period of observation, however, there existed a sudden jump from 83 ± 5 nm to 45 ± 4 nm (Fig. 4b). As proposed in Fig. 4a, when gold nanorod only remained a single touching site with streptavidin, the movement range of biotinylated-DNA-GNR was about 94 nm at most within a semicircle with a radius of its own size (40 nm long-axis plus 7 nm DNA). If biotinylated-DNA-GNR had multiple touching sites with streptavidin, the movement range of nanorods would be confined to a region close to their physical size of 40 nm. This proposed model almost perfectly matched the observed results of D(t) jump. Notable that the observed values of D(t) were not exactly equal to the theoretical model. For instance, the averaged value of single-touch (83 nm) was about 11 nm smaller than its theoretical value 94 nm. It is reasonable since in real situation, the “single touch” nanorods could reach the maximum range of 94 nm but it might not always keep the maximum range, thus the averaged value would be smaller than the theoretical one. Also, the averaged value of D(t) for multi-touch (45 nm) is slightly larger than its theoretical value 40 nm, especially when comparing with the value of static nanorods (35 nm). It could be attributed to the flexible structure of single-strand DNA, which contribute to extra motion range of nanorods. Above discussions could also be proven by the LDP maps and the distribution histograms of D(t) in the whole period of observation (Fig. 4c,e,d,f). When we focused on monitoring the multi-touch, with increasing observation time, several short period recoveries of D(t) from 45 nm to 83 nm occurred (Fig. 4g). These fluctuations of D(t) was presumably attributed to the conversion of touching states between single-touch and multi-touch. This phenomenon seems beyond our general knowledge about the interaction between biotin and streptavidin, since their binding was known as very stable with a binding constant K = 10^13^ M^−1^. The binding constant were usually measured based on large number of molecules, referring to a statistic result. When the molecular interaction was scale down to a few or single recognition events, its random nature and uncertainty could not be neglected. Previous works revealed that hydrogen bonding and van der Waals interactions between biotin and four Trp residues in the binding site of streptavidin have great impact on the streptavidin-biotin dissociation. When the biotinylated-DNA-GNR began to attach to the streptavidin-coated glass slide, the protein-ligand binding contacts might be in the transition state, and Trp 120, Trp 79 contacts and the hydrogen bond between the biotin and streptavidin side chains were greatly weakened^27–29^. The loss of binding contacts might resulted in the opening of the binding pocket, which could be employed to account for the observed conversion of touching modes. However, in the long run, multi-touch dominated most period of time, suggesting that multi-touch is more stable than single-touch, as expected.

## Conclusion

In summary, we studied the transient events of molecular recognition by tracking the motion state of single nanoparticles in spatial-temporal-frequential domain, with a temporal resolution of ~10 ms and a spatial accuracy of ~20 nm. This method enabled us to capture transient moment of molecular encountering and discriminated different binding modes. The transient process of a recognition event cannot be simply considered as static states of on or off, it undergoes stochastic dynamical transformation between different binding modes. This study gain insight into dynamic nature of single recognition events beyond the ensemble characterization of binding constant.

## Methods

### Chemicals

Cetyltrimethylammonium bromide (CTAB), HAuCl_4_·3H_2_O, NaBH_4_, NaCl, glycerol, AgNO_3_ ascorbic acid (AA) were purchased from Sigma-Aldrich, Streptavidin and 5′ Biotin- TCAACATCAGTCTGATAAGCTA-HS-HS-C_6_ 3′ were purchased from Sangon Biotech.

### Instrumentation

The synthesized Gold nanorods were characterized by transmission electron microscopy (JEM-1011 JEOL Ltd., Japan) and UV-vis spectrophotometer (UV-3600, Shimadzu, Japan). Imaging experiments were taken on a Olympus IX 73 inverse microscope, equipped with a dark field condenser (N.A. = 1.28), a 60X/0.75 N.A. objective len and a high speed electron-multiplying charge-coupled device (EM-CCD) with a frame rate up to 100 fps.

### Synthesis of gold nanorods(GNR)

The preparation of Gold nanorods is according to the seed-mediated growth method^30^. Seed solution was prepared by mixing 5 mL, 0.20 M CTAB solution with 5.0 mL of 0.50 mM HAuCl_4_ and rapidly adding 0.60 mL of ice-cold 0.01 M NaBH_4_ under vigorous stirring. After stirring for another 2 min, seed solution was kept at 25 °C at least 1–2 h before use. Briefly, 4.0 mM AgNO_3_ solution, 10.0 mL 1.0 mM HAuCl_4_ was added into 10 mL of 0.20 mL CTAB solution. Then, 140 μL of 0.0788 M AA was used as a mild reducing agent adding into the above mixed solution, dropwise. After the growth solution turned into colorless, 24 μL of the seed solution was added into the growth solution. The growth of Gold nanorods must be maintained at 27–30 °C.

### Functionalization of GNR

250 uL AuNRs was centrifuged at 10000 rpm for 10 min and discarded the supernatant. To this solution, 12.5 μL of 20 μM mPEG-SH was added and followed by vigorous vortexing for 20 s. Before centrifugation and resuspension for three times, the above solution was mixed with 250 μL of 0.01 wt % Tween 20. After that, 20 μL of 10 μM DNA was further added and gradually introduced citrate into the solution to reach a final concentration of 100 mM. The solution was washed by centrifugation at 10000 rpm to remove the unconjugated DNA and redispersed in PBS solution after aging for 1 h.

### Immobilization of streptavidin on glass slide

The glass slide was dropped by a drip of 1.0 mg/mL streptavidin solution (in 20 mM PBS buffer) and kept at 4 °C for 12 h. After washing several times with PBS buffer, glutaraldehyde (1%) was used as a cross-linker to immobilize streptavidin for 1 h at 4 °C. Then, incubating in the same buffer for 3 h at 4 °C and being dried using nitrogen.

## Supplementary information

supporting information
