# Supplementary material for: Revealing transient events of molecular recognition via super-localization imaging of single-particle motion
Source: Sci Rep. 2019 Mar 19;9:4870. doi: 10.1038/s41598-019-41239-5 (PMC6424965; doi:10.1038/s41598-019-41239-5)
Supplement: Supplementary file 1 — supporting information [file 41598_2019_41239_MOESM1_ESM.docx]

**Supplementary Information**

**Revealing transient events of molecular recognition via super-localization imaging of single-particle motion**

Qing-Ying Kong, Fan Yang, Juan Song, Yi-Fan Ruan, Shan-Shan Li, Zhao-Shuai Gao, Bin Kang*, Hong-Yuan Chen, Jing-Juan Xu*

State Key Laboratory of Analytical Chemistry for Life Science and Collaborative Innovation Center of Chemistry for Life Sciences, School of Chemistry and Chemical Engineering, Nanjing University, 163 Xianlin Road, Nanjing 210023, P. R. China


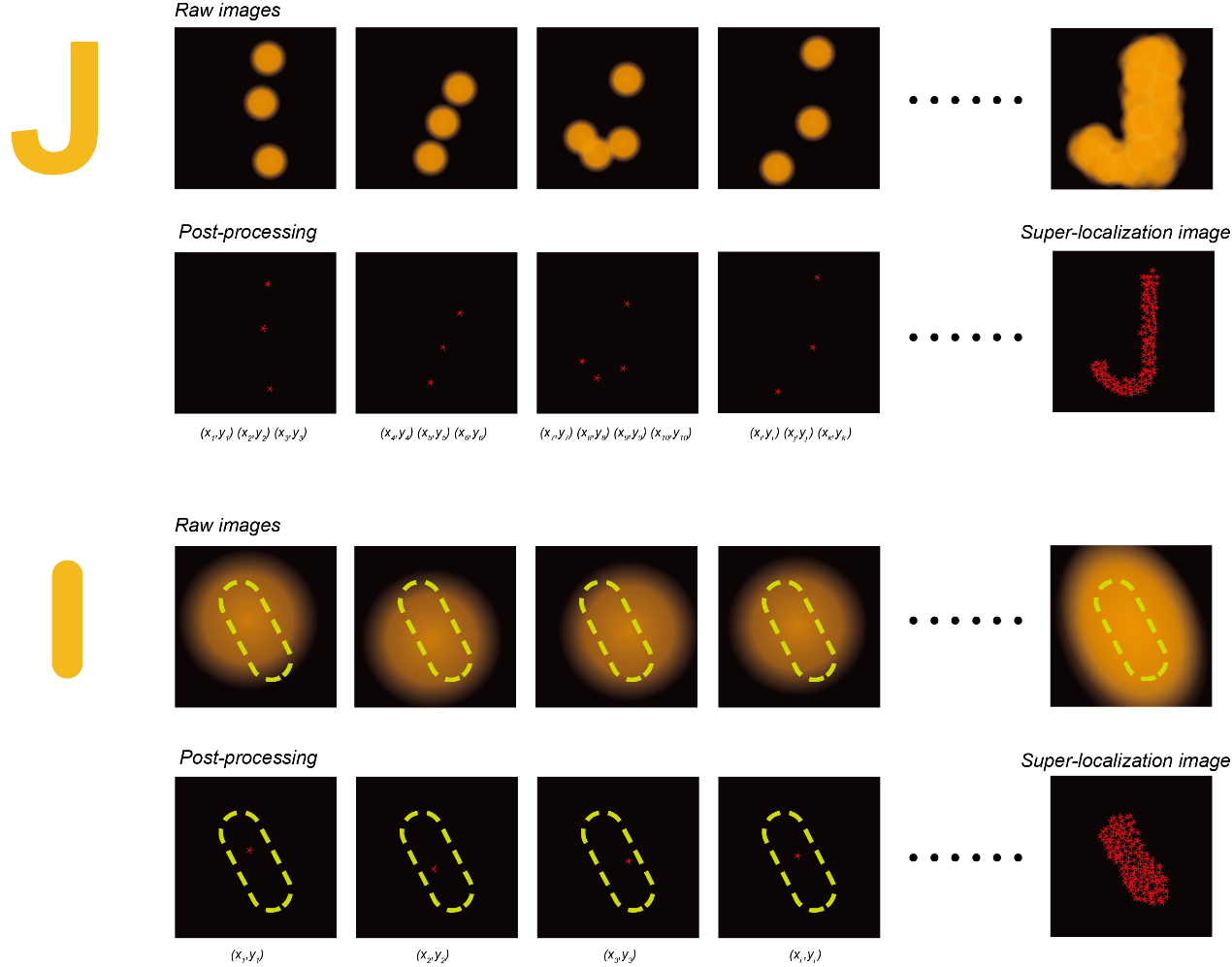


Scheme S1. Principle and working flow of super localization microscopy.

The principle of super localization imaging is based on stochastic collision of incident photons with single GNRs under low flux of incident photons. Once the flux of incident photons were very low, so that only a few scatted photons were collected at one time, each frame of image was shown as stochastic scattering events from different localization of the object. Duo to the wave property of photons, each scatting event was collected as a diffraction limit spot in the final imaging plate (Physical Review Letters 88, 100402, doi:10.1103/PhysRevLett.88.100402 (2002)). After many frame of stacking, the image of the object could be obtained with a diffraction limit resolution, just like a regular image under strong light (see example letter J at the top). If the size of object is close to or even smaller than the diffraction limit, like nanorod, the final stacking image was still a diffraction limit spot as a convolution of object and the point spread function (see example nanorod at bottom). However, by using super localization method, the precision position of each scattering event (x, y) could be extract from the scattering spots in each frame (showing as red stars), then after many frames of localization, the morphology of the object could be finally remapped as a super localization image (showing as red pattern).


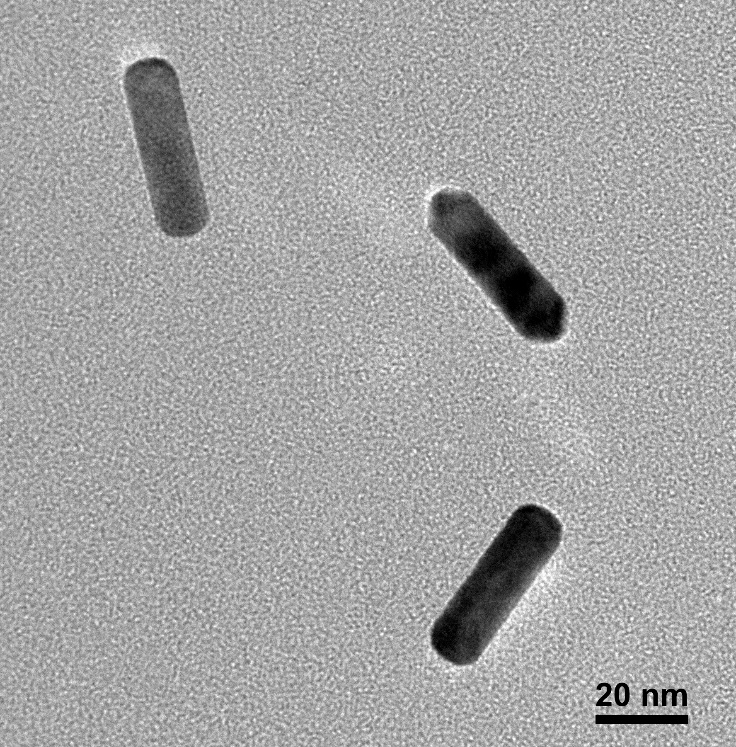


**a**


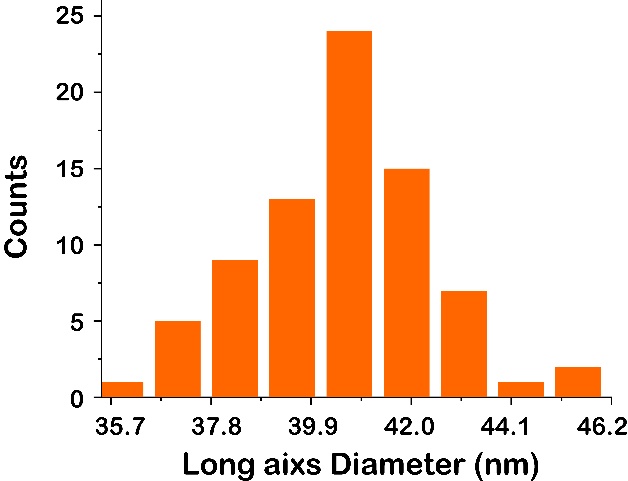

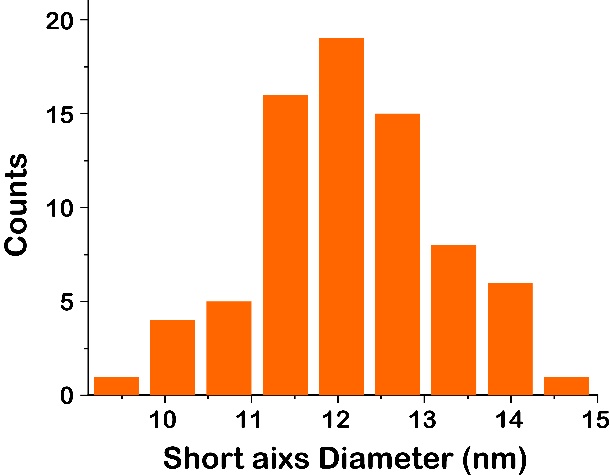


**c**

**b**

**Figure S1**  (a) TEM image of the gold nanorods(GNR). Mean long aixs diameter and short axis diameter of GNR are about (b) 40 nm and (c) 12 nm.

**
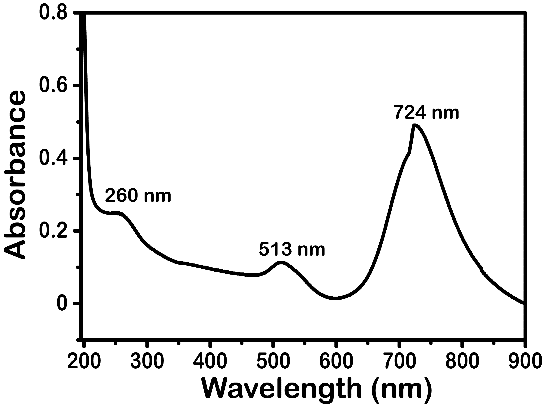
**
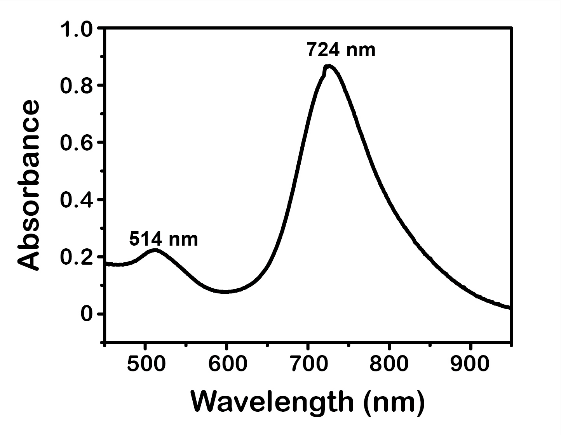


**b**

**a**

**b**

**Figure S2** (A) UV-Vis spectra of the GNR. (B) UV-Vis spectra of the biotinylated-DNA-GNRs.


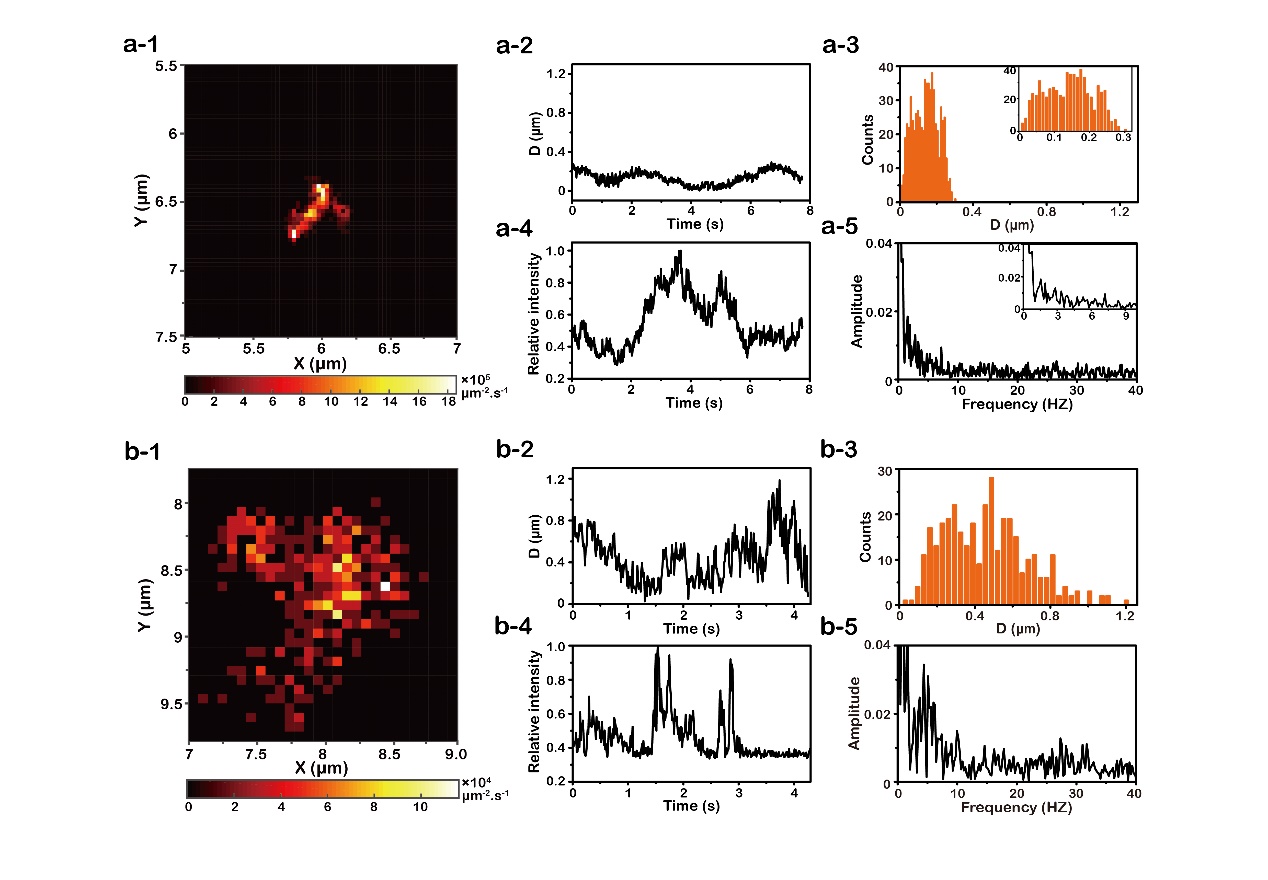


**Figure S3** The Rotational Brownian motion state of a single gold nanorod (GNR) in different media(water/ glycerol). (a-1~a-5) Superlocalization imaging of a GNR rotates in glycerol (a-1) Localization distribution probability (LDP) map. (a-2) Time traces of localization dispersion D(t).(a-3) Histograms of D(t).(a-4) Time trace of optical intensity I(t). (a-5) Fast Fourier transform (FFT) of I(t). (b-1~b5) Superlocalization imaging of a GNR rotates in water. (b-1) LDP map. (b-2) Time traces of D(t). (b-3) Histograms of D(t). (b-4) Time trace of I(t). (b-5) FFT of I(t).


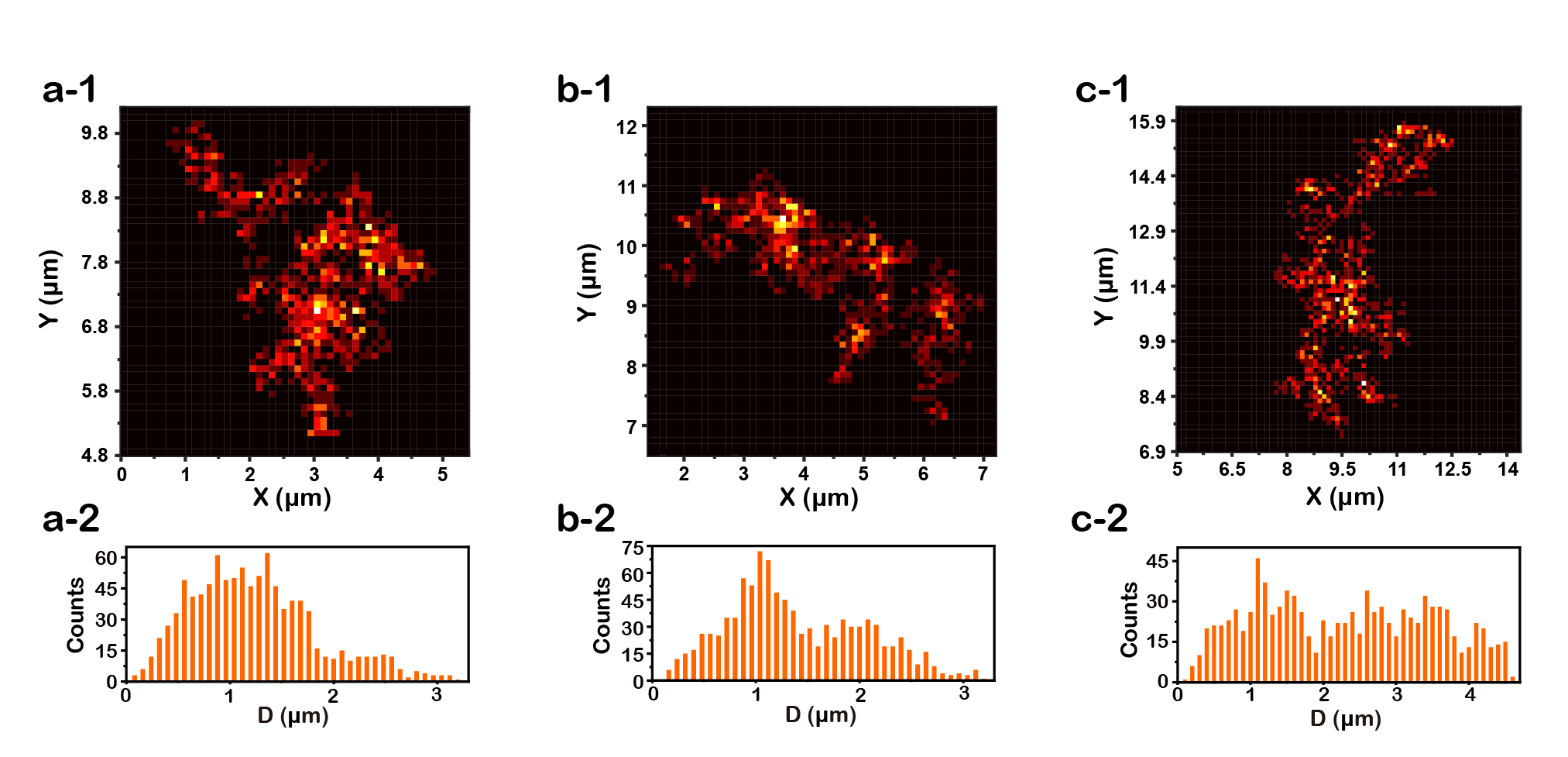


**Figure S4** The Rotational Brownian motion state of a single gold nanorod in water on the streptavidin-coated glass slide.(a-c) Superlocalization imaging of different single GNR rotates in water on the streptavidin-coated glass slide. (a-1, b-1, c-1) Localization distribution probability (LDP) map. (a-2, b-2, c-2) Histograms of D(t).


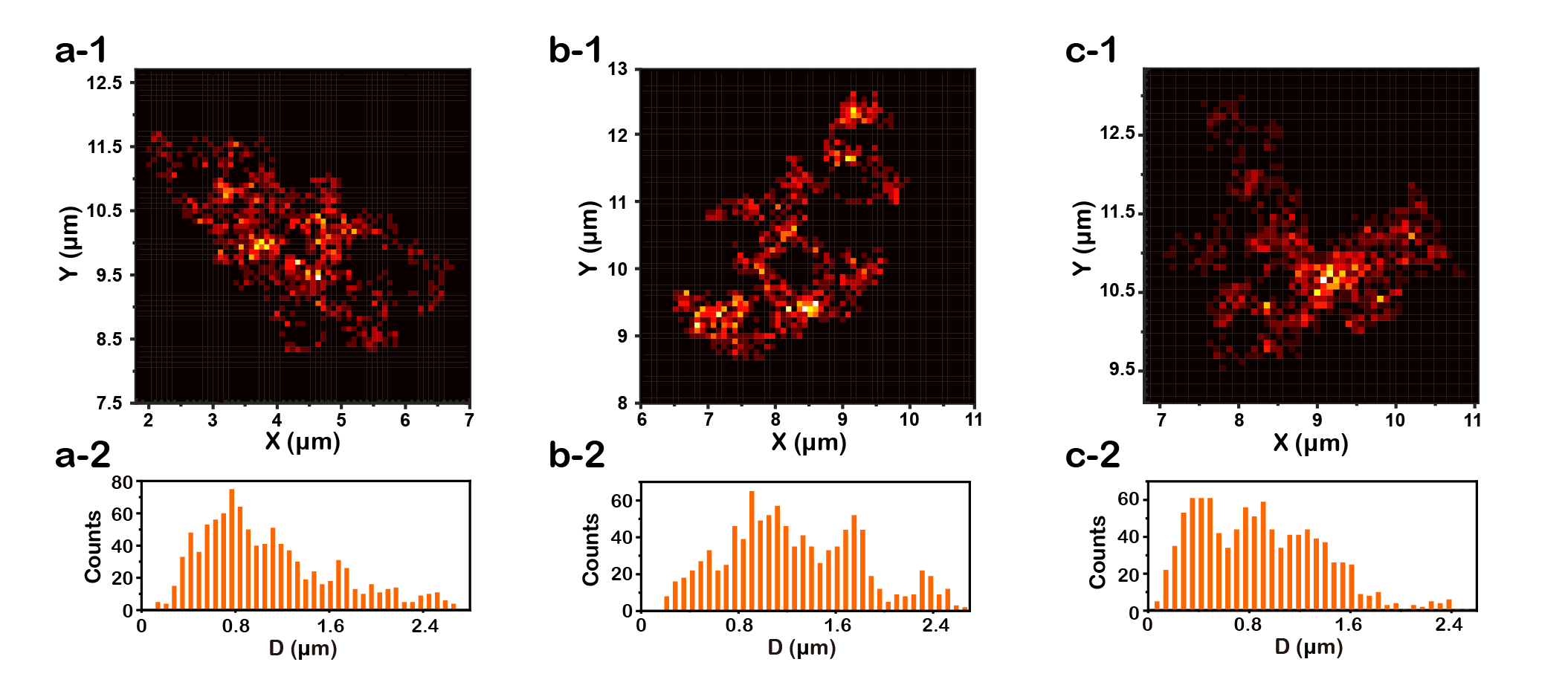


**Figure S5** The Rotational Brownian motion state of a single biotinylated-DNA-GNR in water. (a-c) Superlocalization imaging of different single biotinylated-DNA-GNR rotates in water. (a-1, b-1, c-1) Localization distribution probability (LDP) map..(a-2, b-2, c-2) Histograms of D(t).
